# Location Coding of Tool-Object Pairs Based on Perceptual Grouping: Evidence from Object-Based Correspondence Effect

**DOI:** 10.5334/joc.435

**Published:** 2025-02-26

**Authors:** Usman Jawed Shaikh, Ferdinand Binkofski, Antonello Pellicano

**Affiliations:** 1 Division for Clinical Cognitive Sciences, Department of Neurology, Faculty of Medicine, RWTH Aachen University, Aachen, Germany; 2 Juelich Aachen Research Alliance (JARA)—BRAIN, Juelich, Germany; 3 Research Centre Juelich, Institute of Neuroscience and Medicine (INM-4), Juelich, Germany; 4 Department of Educational Sciences, University of Catania, Catania, Italy

**Keywords:** Simon effect, object-based correspondence effect, affordance, semantic coding, action coding, perceptual grouping

## Abstract

Motor interactions with single, as well as pairs of objects can be automatically affected by visual asymmetries provided by protruding parts, whether the handle or not. Faster and more accurate performance is typically produced when task-defined responses correspond to the location of such protruding parts, relative to when they do not correspond (i.e., object-based spatial correspondence effects). In two experiments we investigated the mechanisms that underlie the spatial coding of tool-object pairs when semantic and action alignment relationships were orthogonally combined. Centrally presented pictures of “active” tools (depicted as potentially performing their proper action) were paired, on one side, to a “passive” object (target of the tool action). We observed S-R correspondence effects that depended on the location of the protruding side of tool-object pairs, and not on the non-protruding side of the tool handle. Thus, results further supported the location coding account of the effect, against the affordance activation one. The effect was only produced when tool-object pairs belonged to the same semantic category or were correctly aligned for action, but with no further interplay. This was not consistent with the idea that action links were coded between tool-object pairs, and that the resulting action direction interacted with response spatial codes. Alternatively, we claimed that semantic relation and action alignment acted, independent from each other, as perceptual grouping criteria; allowing for the basic spatial coding of visual asymmetries to take place. This brought to speculation, at neurocognitive level, about independent processing along the ventral and ventro-dorsal streams.

## Introduction

When we interact with objects in our surroundings, the efficiency of our actions depends on recognizing basic properties like size, location, and orientation in space, as well as relational ones, like their placement in a given context. However, even when no actual motor interaction is required, the location of a perceived object can influence our motor behavior in an automatic way. This has been widely demonstrated in stimulus-response (S-R) spatial correspondence tasks where visual stimuli are presented, on the left or on the right in the participants’ visual field, while left and right button press responses are mapped to a nonspatial feature of them (e.g., color, shape, or size). Within such S-R *dimensional overlap* (Kornblum et al., 1990) stimuli automatically trigger responses from their same side which ultimately speeds up spatially corresponding responses and slows-down non-corresponding ones (i.e., *Simon effect*, Simon, 1990). Automatic response codes can also be generated by objects themselves, based on the location of specific components (e.g., Anderson et al., 2002; Cho & Proctor, 2010; Pellicano & Binkofski, 2021). In a typical experimental task, common-use objects (e.g., kitchen tools such as cups or mugs) are presented in the center of the visual field, but with a left- or rightwards protruding part. Since the protruding portion frequently consists in the graspable side, that is the *handle*, such effects have been named *handle orientation effects* (see Tucker & Ellis, 1998) or *handle-to-hand correspondence effects* (Pellicano & Binkofski, 2021; Pellicano, Koch & Binkofski, 2017). According to the most recent *location coding account* (Cho & Proctor, 2010; Proctor & Miles, 2014), protruding handles become perceptually salient to the observer as they create *visual asymmetry* within the stimulus display. The location of such salient parts induces the spatial coding of the object stimuli, and in turn a dimensional overlap with the response sets. Consequently, task-designated responses result faster and more accurate when they are performed on the same side as this protruding part, whether the handle or not, compared to when they are performed on the opposite side. Thus, an *object-based correspondence effect* or *object-based Simon effect* is provided (Proctor & Miles, 2014), that shares the same mechanisms as the original Simon effect.

To note, the handle-to-hand correspondence effect has been originally attributed to a different grasping affordances mechanism. According to this *affordance activation account*, an object tool promotes in the observer the activation of motoric actions which are consistent with its identity and proper use (Bub & Masson, 2010; Goslin et al., 2012). Indeed, an object generates grasping actions towards its graspable side whenever its functional meaning is assessed. These actions involve the left or the right hand depending on the left- or rightward orientation of the handle (variable affordances; see Borghi & Riggio, 2015; Pellicano, Borghi, et al., 2017; Pellicano et al., 2011). Consequently, the responses are faster and more accurate when the responding hand is aligned with the orientation of the objects graspable side (handle), relative to when it is not. In the wake of this hypothesis, other authors investigated the motor properties of tools within visual scenarios that included other objects. Using S-R compatibility paradigms, they provided evidence of response facilitation when the responding hand was aligned with the handle of a tool paired to a second object, if tool and object were co-located for action and functionally linked (e.g., Borghi et al., 2012). Thus, tools manipulability would be better activated when their proper function is made more explicit within a visual scene (see also Buxbaum, 2001; Yoon et al., 2010).

The mechanisms at play which are responsible for handle-to-hand correspondence effects are still subject of debate. More recent contributions have proposed that affordance and spatial coding may not be mutually exclusive but, to some extent, contribute both to correspondence effects depending on the manipulation of stimuli, responses, and task features at hand (see for example Bub, Masson, & van Noordenne, 2021).

In sum, according to the location coding account any protruding component of an object, not necessarily its handle, confers visual asymmetry and is responsible of a lower-level, abstract spatial coding of the stimulus. According to the affordance activation account, the handle itself drives higher-level processing of the object tool finalized to properly grasp and use it. If, however, as in most cases, the graspable and salient portions coincide, it remains impossible to identify which process is responsible for the handle-to-hand correspondence effect. Several authors have addressed this issue (Azaad et al., 2019 for a meta-analysis; Cho & Proctor, 2011, 2013; Kourtis & Vingerhoets, 2015; Pellicano et al., 2010, 2017; 2019; Pellicano & Binkofski, 2021). Among them, Pellicano Koch & Binkofski (2017) investigated the contribution of stimulus features, while employing button press responses. They utilized pictures of single tools, as well as tools paired to an object. Single tools were creamers and teapots with a graspable portion that was not visually salient (providing no lateral asymmetry to the object) and a salient, non-graspable portion (i.e., the spout) on the opposite side. Paired tools and objects included the same creamers and teapots with a second target object placed close to their spout (see below for a detailed description). Results supported the location coding account since observed correspondence effects, for single tools, depended on the orientation of their visually salient spout and never on the orientation of their non-salient graspable side. Most crucially, Pellicano, Koch, & Binkofski, 2017 proposed an *action coding account* of object-based correspondence effects that further qualified the original location-coding account when *pairs* of objects were displayed, beyond single ones. According to it, a direction code was created when a plausible action was depicted from the tool to the object, relative to when no action was possible (see Pellicano et al., 2017- Experiment 4). Such action direction codes are determined by higher-level semantic and action processes, rather than lower-level processes responsible for basic visual asymmetries in the stimulus configuration (see also Kourtzi & Kanwisher, 2000).

## The present study

We aimed to follow-up the investigation of Pellicano et al. (2017) to test the contribution of semantic relation and alignment information in the processing of manipulable objects, and contextually to further test the *action coding account* of the object-based correspondence effect. In their Experiment 4, stimuli were pairs of objects consisting of an “active” *tool* placed close to a “passive” *object*. Tool-object pairs could share a semantic relation, that is, they belonged to the same “kitchenware” category, together with an action relation, since they were typically used together (e.g. “creamer + cup” or “teapot + cup”); or they did not share any semantic-action relation (e.g. “creamer + golf ball” or “teapot + golf ball”). Participants discriminated whether paired tools and objects belonged to the same semantic category or to different ones by pressing a left or a right button.

A correspondence effect was observed (faster responses when the protruding side of tool-object pairs corresponded to the response location, relative to when they did not correspond) when tool-object pairs shared a semantic-action relation (e.g. creamer + cup), but not when they shared no relation at all (e.g., creamer + golf ball). Results suggested that participants coded the direction of a plausible action link between tool and object (i.e., pouring leftward or rightward), rather than coding the basic visual asymmetry of the objects pair (according to which, the same correspondence effect would have been observed in both the conditions).

In the original study, semantic and action relations were basically integrated to each other within the employed tool-object pairs. The tool was also presented in upright and in upside-down orientations, relative to the side object that was always upright. Nevertheless, this had no effect on performance; for example, hampering the action relation while not affecting the semantic one. In the present study, we deepened the relative weight of semantic relation and action alignment in tool-object pairs. Different tool-object arrangements were implemented to provide orthogonal combination of semantic and action relations. We selected different side objects and also manipulated the horizontal orientation of the central tool, so that it resulted in pointing at the object or away from it (while in the original study the tool was always pointing at the object, even when it was upside-down). Furthermore, a comprehensive investigation was planned across two experiments, with tool-object semantic relation and alignment being the task-relevant and task-irrelevant features in Experiment 1; vice versa in Experiment 2.

Specifically, semantic relation and action alignment features of tool-object pairs were manipulated as follows: in the same category and action aligned condition (e.g., upright creamer pointing at an upright glass or upright hammer pointing at an upright nail) a plausible and available action was depicted. In our rationale, a *plausible* action holds to the semantic relation and involves an active tool and a passive object that belong to the same context (kitchen or garage) and are typically used together (functional relation, Borghi, Flumini, Natraj, Wheaton, 2012). Conversely, an *implausible* action involves tools and objects that belong to different contexts and are not typically used together. An *available* action, instead, holds to the action alignment: it is the one that is viable from the tool to the object, both displayed in their own canonical orientation, and arranged to each other so that there are no obstacles to its execution. Then, an action becomes *unavailable* when, for instance, the spatial orientation of tools and objects (upside-down or pointing away from each other) or their state (e.g. broken or hidden) hampers any action to be performed. Therefore, in the same category and action misaligned condition, an action is plausible but unavailable; in the different category and action aligned, an action is implausible but available, and in the different category and action misaligned condition, action is implausible and unavailable.

According to our most restrictive hypothesis, if the coding of an action direction depended on the combination of both semantic relation and alignment between tool and object, then a correspondence effect should be only produced when the two objects belonged to the same category and were correctly aligned (plausible and available action link). Instead, no correspondence effect should be produced in the other three conditions: when the semantic relation was given, but the two objects were misaligned; when objects were semantically unrelated and aligned, or misaligned.

Results from both the experiments did not support our action coding account of object-based correspondence effect. Nevertheless, results offered a clear scenario that was consistent with an alternative explanation grounded on perceptual grouping mechanisms (Bar, 2004; Gronau et al., 2008; Xu & Chun, 2007).

## Experiments

### Method

#### Participants

Forty-four students from RWTH Aachen University participated in the study. Twenty-two students took part in Experiment 1 (11 females, 11 males; mean age 24.34 years; *SD* 3.30 years), and twenty-two in Experiment 2 (15 females, 7 males; mean age 24.70 years; *SD* 5.22 years). All of them gave informed consent and received 10 Euros for their participation. All participants were right-handed according to the Edinburgh Inventory of Handedness (Oldfield, 1971): +78/100, SD 18.01 in Experiment 1, and +76/100, SD 14.45 in Experiment 2. All had normal or corrected-to-normal vision, normal color vision, and were naïve as to the purpose of the experiment. According to a power analysis performed with G*Power (v. 3.1.9.7, Faul et al., 2007), a medium effect size f = 0.25 (Cohen, 2013) – for an *F* statistical test employing our 2×2×2×2 model (one between-participants and three repeated-measures within-participants factors; see below), associated to 95% power, at a significance level of α = .05 – would require a sample size of N = 24. Consequently, the acquired sample size of N = 44 was deemed sufficient for the assessment of the study hypothesis.

#### Materials

Participants comfortably seated facing a 17” PC monitor (1024 × 768 screen resolution) driven by a 3-GHz PC. Stimulus presentation, response timing, and data collection were controlled by the E-Prime Professional v2.0 software (http://www.pstnet.com). Participants viewed the stimuli from an eye distance of 57 cm with the help of a chin-rest. A button box (PST serial response-box) was connected to the PC with its left- and rightmost buttons (1’ or ‘5’) being 15 cm apart and 20 cm from the screen. Both the screen and the button box were centered on the body midline of the participant. The experiment had a black central fixation cross (0.4 × 0.4 degrees of visual angle^1^), and the target stimulus presented on a white background.

**Figure 1:**
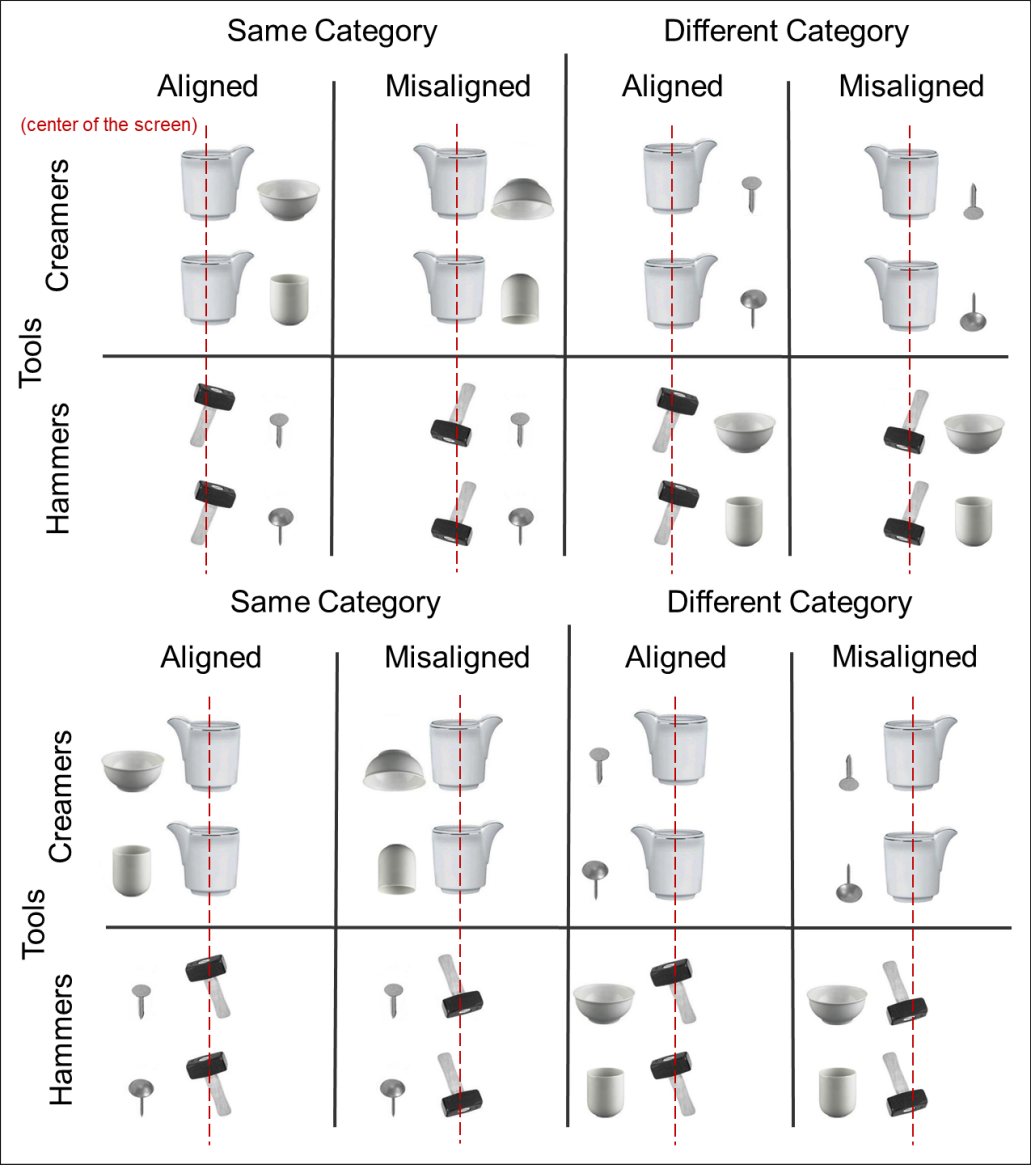
Diagram of sample visual stimuli (tool-object pairs) protruding to the right (upper panel) and to the left (lower panel) presented in Experiment 1 and Experiment 2.

Target stimuli consisted of randomly presented color pictures of tool-object pairs. Tools consisted of three different *creamers* (ranging from 6.0**°** to 7.6**°** wide × 7.3**°** to 7.8**°** high) and three different *hammers* (ranging from 3.8**°** to 4.9**°** wide × 7.0**°** to 7.4**°** high), each paired to one symmetrical object with no jutting portions: one *cup* (6.5**°** wide × 4.5**°** high), or one *glass* (4.0**°** wide × 5.6**°** high), or two different *nails* (ranging from 2.0**°** to 2.7**°** wide × 4.0**°** to 4.3**°** high). The three creamer + object pairs ranged from 10.5**°** to 14.9**°** wide and 7.3**°** to 7.8**°** high; the three hammer + object pairs ranged from 9.5**°** to 13**°** wide × 7.0**°** to 7.4**°** high. The body of the tool was centered on the screen and along the body midline, while the object was located on its left or right, therefore the tool-object pair could protrude to the left or right depending on the location of the object. Tool-object pairs had eight different configurations: (i) They belonged to the same semantic category (kitchenware and working tools, i.e., creamer + cup/glass; hammer + nail), or to different categories (i.e., creamer + nail; hammer + cup/glass); (ii) they were aligned to each other (i.e., upright creamer with its spout pointing towards the upright object, and upright hammer with its head tilted towards the upright object), or misaligned (i.e., upright creamer with its spout oriented away from the upside-down object, and conversely, upside-down hammer with its head tilted away from the upright object); (iii) they were left- or rightward oriented. The tool-object arrangements for the misaligned condition were selected as they were most clearly judged as misaligned by a group of six naïve test persons.^2^ The misaligned condition was expected to hamper or disrupt any action link between tool and object (Figure 1). Including the three different creamers and hammers, each paired to four objects, a total of 96 stimuli were administered in each experiment. These stimuli were repeated six times across the experiment for a total of 576 trials.

#### Procedure

The experiments took place in the Behavioural Laboratory of the Division for Clinical Cognitive Sciences, Department of Neurology, Faculty of Medicine, RWTH Aachen University (https://www.ccs.rwth-aachen.de/index.php/en/research/behavioural-laboratory) which was dimly lit and noise attenuated. At the beginning of each trial, the fixation cross was presented for 1000 ms together with a warning high pitch tone of 100 ms, followed by the target stimulus, which remained on screen for a maximum of 1000 ms or until a response was produced. Participants rested their left and right index fingers on the left and right response buttons for the whole duration of the experiment. In Experiment 1, half the participants had to discriminate whether each tool-object pair belonged to the same or different semantic category, by pressing either the left- or the right button with their left or right index finger, respectively. The other half had opposite stimulus-response mapping. Both the stimulus leftward-rightward orientations and the reciprocal alignment-misalignment were task-irrelevant and had to be ignored. In Experiment 2, opposite to Experiment 1, tool-object alignment/misalignment for action was task-relevant, while semantic relation was task-irrelevant. Half the participants pressed the left button when tools and objects were aligned, and the right button when they were misaligned; the other half had opposite S-R assignments. At correct responses, a blank screen was displayed for 800 ms. If an incorrect or no response occurred, the words “FALSCH” (wrong) or “FEHLT” (missing) were provided for 1000 ms together with a 300 ms low pitch tone. Each experiment was divided in 3 blocks of 192 trials each and separated by short resting breaks. Experimental trials were preceded by 10 training trials. The total duration of each experiment was 45 minutes.

#### Design

The original reaction time distributions from each participant significantly deviated from normality, as assessed by the Shapiro-Wilk test, *p*s < .001. The Box-Cox procedure (Box & Cox, 1964) was applied to compute normal distributions. We analyzed normalized RTs of correct responses (from now on RTs) with a linear mixed model (LMM) and counts of response accuracies with a generalized linear mixed model (GLMM). All these analyses were performed in SPSS (IBM, U.S.A.). The statistical model included the fixed factors: *Experiment* (Experiment 1 vs. Experiment 2) as between-participants; *Category* (same vs. different), *Action* (aligned vs. misaligned), and *Correspondence* (location of the second object: corresponding vs. non-corresponding to the response location) as within-participants. Furthermore, *Participants* and *Stimuli* were included as random factors. The *superb* package (Cousineau et al., 2021) in the R statistical software (R Core Team, 2016) – designed to calculate 95% confidence intervals through the Cousineau-Morey decorrelation method (Cousineau, 2005; Morey, 2008) – was employed to draw error bars for graphic illustration of between-conditions differences. Intraindividual effects were computed between conditions of interest (e.g. non-corresponding minus corresponding); post-hoc one-sample *t*-tests (Bonferroni-corrected) were then applied to such effects, including the calculation of standardized Cohen’s *d_z_*s effect size estimates and confidence intervals (CIs) (all performed in SPSS). Indeed, beyond the *p* value, when these CIs included zero, then the effect was considered as non-significant.

In sum, on the basis of our original action coding hypothesis (Pellicano et al., 2017), a significant interaction between Category, Action, and Correspondence would be produced, irrespective of Experiment (i.e., whether tool-object alignment/misalignment for action or semantic relation were relevant for the task). Specifically, a correspondence effect should be observed in the same category and aligned condition (providing plausible and available action links), but not in the other three conditions: same category and misaligned, different category and aligned, or misaligned.

### Results

For each participant, omitted responses (0.9% in Experiment 1 and 0.5% in Experiment 2) were excluded from the analyses. Normalized RTs lower than median minus 2.5*MAD (median absolute deviation) and higher that median plus 2.5*MAD were considered outliers (see Leys et al., 2013) and discarded (0.51% and 0.55% in Experiment 1 and 0.51% 0.52% in Experiment 2).

#### RTs

The main effect of *Experiment* was significant *F*(1, 43) = 15. 419, *p* < .001, with faster RTs in Experiment 2 relative to Experiment 1 (534 vs. 593 ms). The main effects of *category, action*, and *correspondence* turned significant, *F*(1, 92) = 104.269, *p* < .001, *F*(1, 92) = 65.627, *p* < .001, and *F*(1, 24688) = 26.048, *p* < .001; with shorter RTs when the two objects belonged to the same category relative to when they belonged to different categories (553 vs. 574 ms), when they were aligned relative to misaligned (555 vs. 572 ms), and when the salient side of stimuli corresponded to response locations relative to when they did not correspond (561 vs. 567 ms). Also, they interacted with Experiment: *Category × Experiment, F*(1, 24668) = 146.865, *p* < .001, *Action × Experiment, F*(1, 24668) = 214.778, *p* < .001, and *Correspondence × Experiment, F*(1, 24668) = 12.496, *p* < .001. The effect of Category was significant in experiment 1 (same = 575 vs. different = 611 ms), *t*(21) = 7.459, *p* < .001, with *d_z_* = 21.97, 95% CI = [0.95–2.22], and in experiment 2 (530 vs. 538 ms), *t*(21) = 4.577, p < .001, *d_z_* = 8.63, 95% CI = [0.44–1.42] (Bonferroni-corrected alpha = .025), but larger in experiment 1. The effect of Action was not significant in experiment 1, *t*(21) = 0.102, *p* = .920, *d_z_* = 9.13, 95% CI = [–0.40–0.44], and significant in experiment 2, *t*(21) = 5.833, *p* < .001, *d_z_* = 24.83, 95% CI = [0.67–1.80]. The effect of correspondence was not significant in experiment 1, *t*(21) = 0.658, *p* = .518, *d_z_* = 9.10, 95% CI = [–0.28–0.56], and significant in experiment 2, *t*(21) = 3.061, *p* = .006, *d_z_* = 14.02, 95% CI = [0.18–1.07]. The *category × action* interaction was not significant, *F*(1, 92) = 2.332, *p* = .130; however, *category × action × experiment* reached significance, *F*(1, 24688) = 6.230, *p* = .013. Thus, in experiment 1, we observed nonsignificant differences between aligned and misaligned trials in both same, *t*(21) = 2.179, *p* = .041, *d_z_* = 12.08, 95% CI = [–0.90 – –0.02] and different category conditions, *t*(21) = 1.678, *p* = .108, *d_z_* = 14.58, 95% CI = [–0.08–0.78]. In experiment 2, we instead observed significant differences between aligned and misaligned trials in both same, *t*(21) = 6.183, *p* < .001, *d_z_* = 23.67, 95% CI = [–1.89 – –0.73] and different category conditions, *t*(21) = 4.789, *p* < .001, *d_z_* = 29.92, 95% CI = [–1.53 – –0.49] (corrected alpha level = .012).

**Figure 2:**
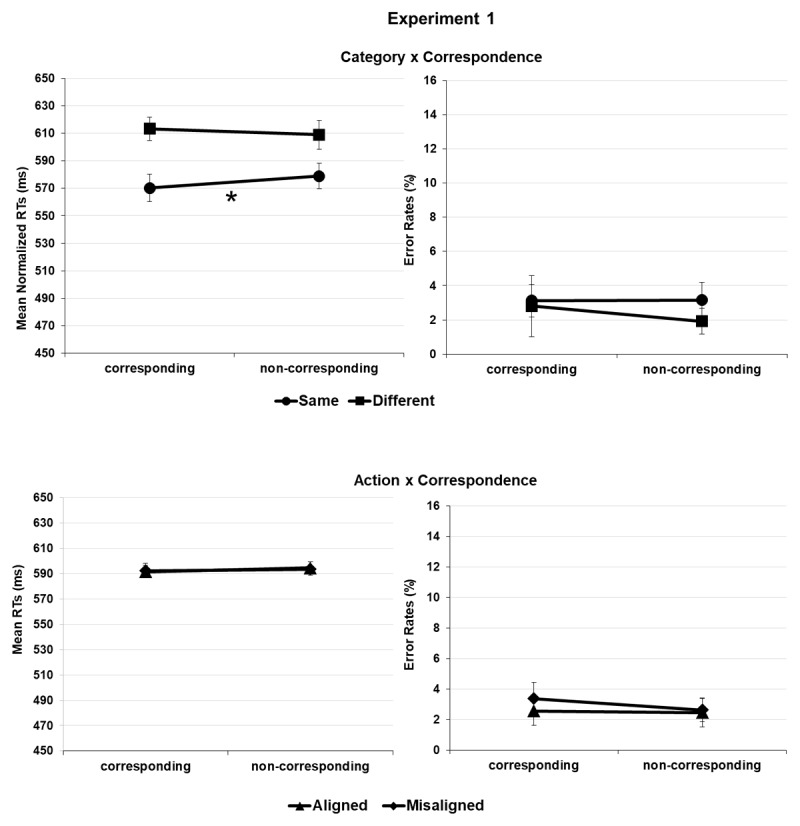
Experiment 1. Salient portion-to-response correspondence effect (e.g., spout plus second object location-to-response position corresponding vs. non-corresponding pairings) in the task-relevant same and different category conditions (upper panel) and in task-irrelevant alignment and misalignment for action (lower panel); mean normalized reaction times (RTs) and error rates (ERs).

**Figure 3:**
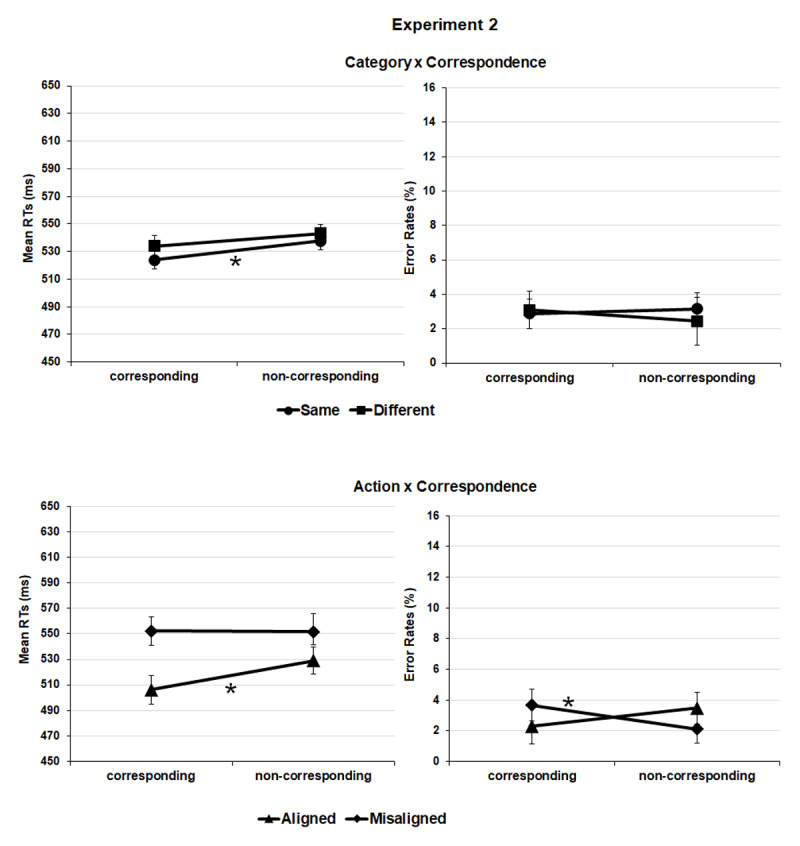
Experiment 2. Salient portion-to-response correspondence effect (e.g., spout plus second object location-to-response position corresponding vs. non-corresponding pairings) in the task-relevant alignment and misalignment for action (lower panel) and in task-irrelevant same and different category conditions (upper panel); mean normalized reaction times (RTs) and error rates (ERs).

Crucially, the interaction between *correspondence* and *category* was significant, *F*(1, 24688) = 13.193 *p* < .001, showing a salient side-to-response correspondence effect when the object pairs belonged to the same category (9 ms), *t*(43) = 4.705, *p* < .001, *d_z_* = 13.02, 95% CI = [0.37–1.04] but not when they belonged to different categories (1 ms), *t*(43) = 0.526, *p* = .301, *d_z_* = 14.95, 95% CI = [–0.22–0.37] (corrected alpha level = .025). This result was further qualified by the second-level *correspondence × category × experiment* interaction, *F*(1, 24688) = 4.671 *p* = .031 according to which the correspondence effect showed up in the same category condition of experiment 1, *t*(21) = 2.814, *p* = .010, *d_z_* = 12.21, 95% CI = [0.14–1.05], and of experiment 2, *t*(21) = 3.790, *p* < .001, *d_z_* = 13.80, 95% CI = [0.32–1.28], indeed either when semantic relation was task-relevant or not; but not in the different category condition of both experiment 1, *t*(21) = 1.915, *p* = .069, *d_z_* = 11.67, 95% CI = [–0.84–0.03], and experiment 2, *t*(21) = 2.130, *p* = .045, *d_z_* = 15.72, 95% CI = [0.31–1.24] (corrected alpha level = .012) (Figures 2 and 3, upper panels).

*Correspondence* also interacted with *action, F*(1, 24688) = 24.859, *p* < .001, showing a protruding side-to-response correspondence effect when the object pairs were aligned for action (11 ms), *t*(43) = 3.691, *p* < .001, *d_z_* = 19.32, 95% CI = [0.24–0.87], but not when they were misaligned for action (0 ms), *t*(43) = 0.163, *p* = .871, *d_z_* = 13.13, 95% CI = [–0.32–0.27] (corrected alpha level = .025). Similar to the earlier report, this interaction was further qualified by the second level *correspondence × action × experiment* interaction, *F*(1, 24688) = 17.450, *p* < .001. The correspondence effect was only significant in the aligned condition of experiment 2, *t*(43) = 4.311, *p* < .001, *d_z_* = 20.92, 95% CI = [0.40–1.36] and not of experiment 1, *t*(21) = 0.802, *p* = .432, *d_z_* = 13.25, 95% CI = [–0.25–0.59] or in the misaligned conditions of experiment 1, *t*(21) = 0.138, *p* = .891, *d_z_* = 9.84, 95% CI = [–0.39–0.45], and experiment 2, *t*(21) = 0.275, *p* = .786, *d_z_* = 15.98, 95% CI = [–0.48–0.35] (corrected alpha level = .012) (Figure 2 and Figure 3, lower panels). No further *correspondence* × *category* × *action* or *correspondence* × *category* × *action × experiment* interactions were significant, *F*(1, 24688) = 1.797, *p* = .180 and *F*(1, 24688) = 2.985, *p* = .084, respectively (Tables 1 and 2).

**Table 1:** Experiment 1: Salient portion-to-response correspondence effect for RTs (milliseconds) and ERs (percentages of errors) in same and different semantic categories and aligned and misaligned conditions.


CATEGORY	ACTION	CORRESPONDENCE			CORRESPONDENCE EFFECT	
	
			RTS	ERS	RTS	ERS

Same	Aligned	Corresponding	569	2.5	6	0.4

		Non-corresponding	575	3.0		

	Misaligned	Corresponding	573	3.7	11	–0.4

		Non-corresponding	584	3.3		

Different	Aligned	Corresponding	614	2.6	0	–0.6

		Non-corresponding	614	2.0		

	Misaligned	Corresponding	613	3.0	–10	–1.1

		Non-corresponding	603	1.9		


**Table 2:** Experiment 2: Salient portion-to-response correspondence effect for RTs (milliseconds) and ERs (percentages of errors) in same and different semantic categories and aligned and misaligned conditions.


CATEGORY	ACTION	CORRESPONDENCE			CORRESPONDENCE EFFECT	
	
			RTS	ERS	RTS	ERS

Same	Aligned	Corresponding	501	2,1	23	1.5

		Non-corresponding	524	3,6		

	Misaligned	Corresponding	547	3,6	1	–0.9

		Non-corresponding	548	2,7		

Different	Aligned	Corresponding	511	2,5	19	0.9

		Non-corresponding	530	3,3		

	Misaligned	Corresponding	556	3,7	–2	–2.2

		Non-corresponding	554	1,5		


#### ERs

No main effect of action or correspondence resulted significant, *F*s(1, 24821) < 1. The main effect of *Category* was significant, *F*(1, 24821) = 5.022, *p* = .025; overall performance was more error prone in the same (3.1%), relative to different category condition (2.6%). *Category* interacted with *Correspondence F*(1, 24821) = 5.816, *p* = .016, with no significant effect in the same, *t*(43) = 0.529, *p* = .600, *d*_z_ = 1.98, 95% CI = [–0.22–0.37] (3% vs. 3.2%), as well as in the different category condition, *t*(43) = 1.1614, *p* = .114, *d*_z_ = 3.18, 95% CI = [–0.54–0.06] (3% vs 2.2%); significance was instead due to smaller percentage of errors for S-R non-corresponding trials in different relative to same category condition (2.2% vs 3.2%), *t*(43) = 2.954, *p* = .005, *d*_z_ = 2.20, 95% CI = [0.13–0.75]. Thus, performance appeared to be less error prone when the trials shared the “negative” value of both the variables.

The *correspondence* × *action* interaction was significant *F*(1, 24821) = 16.965, *p* < .001. We observed a cross over between the effects functions: a nonsignificant positive correspondence effect (i.e., lower percentage of errors in the corresponding relative to the non-corresponding trials) for aligned tool-object pairs (2.4% vs. 3%), *t*(43) = 1.391, *p* = .171, *d*_z_ = 2.56, 95% CI = [–0.09–0.51]; and a significant negative correspondence effect for misaligned tool-object pairs (3.5% vs. 2.4%), *t*(43) = 3.351, *p* = .002, *d*_z_ = 2.28, 95% CI = [–0.82–0.19]. The significant *correspondence × action × experiment* interaction, *F*(1, 24821) = 6.996, *p* = .008 specified that such cross over pattern was produced in experiment 2, only. Thus, no significant correspondence effect was observed in the aligned and misaligned conditions of experiment 1, *t*s(21) ≤ 1.702, *p*s ≥ .104 (Figure 2, lower panel), whereas in experiment 2, a nonsignificant positive effect was produced in the aligned condition, *t*(21) = 1.982, *p* = .061, *d*_z_ = 2.76, 95% CI = [–0.02–0.85], and a significant negative effect in the misaligned one, *t*(21) = 2.970, *p* = .007, *d*_z_ = 2.44, 95% CI = [–1.09–0.17] (Figure 3, lower panel).

## General discussion

In the present study, we investigated the mechanisms that underlie the spatial coding of tool-object pairs when a semantic and an action alignment relationship were manipulated between them and mapped to button press responses. We followed-up the original investigation in Pellicano, Koch and Binkofski (2017) while providing a clear separation between the semantic and the action alignment relations. Pictures of “active” tools (i.e., creamers and hammers depicted as potentially performing their proper action), were employed as paired with a second “passive” object (i.e., the target of tool actions). The presence/absence of semantic relations and of alignments for action were orthogonally combined within the tool-object pairs to define four experimental conditions (Figure 1). Two experiments were conducted on the same stimulus materials. In experiment 1, the semantic relation between tools and objects was task-relevant, whereas in experiment 2, that was the action relation.

The tool-object semantic and alignment relations overall affected performance, as well as the correspondence between the tool-object lateral protrusion and the response location. Performance was faster in experiment 2 relative to experiment 1, suggesting that overt alignment discrimination between tools and objects resulted easier relative to the overt semantic discrimination. Interactions clarified that the effect of semantic relations was significant either when category discrimination was task-relevant (in experiment1) or potentially neglectable as task-irrelevant (in experiment 2): RTs were faster when tool-object pairs belonged to the same semantic category, relative to when they were from different categories (Bach et al., 2005; Roberts & Humphreys, 2010). Instead, the effect of action alignment was significant when this feature was task-relevant and overtly processed (experiment 2), but not when it was task-irrelevant (experiment 1): in the first condition we observed faster performance if tools and objects were correctly aligned relative to when they were misaligned.

Crucially, correspondence interacted with category and experiment. In experiment 1, the S-R correspondence effect was produced when the tool-object pairs belonged to the same category, and not when they were semantically unrelated. This result was similar to Pellicano et al. (2017) – Experiment 4; however different from the original experiment, here we provided a clearer distinction between semantic and action alignment features of stimuli. The same evidence of significant correspondence effect in the same category condition, but not in the different category condition was observed in experiment 2. Thus, through the two experiments, the spatial coding of the tool-object pairs was affected by their semantic features, irrespective of being overtly or covertly processed.

Correspondence interacted with action and experiment too. The S-R correspondence effect was produced when tools and objects were correctly aligned, and not when they were misaligned, but only when the action alignment feature was relevant for the task completion (experiment 2); not when it was irrelevant for it (experiment1).

No further interplay was observed between same category and action aligned conditions in determining the correspondence effect, as indicated by the nonsignificant category × action × correspondence and category × action × correspondence × experiment interactions. This allowed us to conclude that, in experiment 1, spatial codes were apparently assigned exclusively as a function of semantic links between tools and objects. Thus, the same spatial coding mechanisms affected the processing, for example, of an upright creamer with its spout pointing at an upright cup, or the same creamer pointing away from an upside-down cup. In experiment 2, the same spatial coding mechanism, based on semantic information, took place. Here, however, spatial codes were also (and independently) based on the alignment between tools and objects, with a spatial coding mechanism that affected the processing, for example, of either an upright creamer with its spout pointing at an upright cup, or the same creamer pointing at a nail.

Before entering the core discussion of our results, we discuss here the general contribution of our investigation in the light of existing related studies. Our approach differs from most of the existing contributions since it focuses on the strict control of perceptual asymmetries within manipulable stimuli to remove the overlap, and the confusion, between the effects of spatial coding mechanisms and the effects of affordances activations. In our studies, we observed S-R correspondence effects driven by the right-leftward protruding side of single tools, as well as of tool-object pairs. Indeed, within the ongoing debate on the nature of motor facilitation effects toward tools, we basically supported the location coding account for object-based correspondence effects (Pellicano et al., 2021; 2019; Cho & Proctor, 2013; Proctor & Miles, 2014).

Most of this evidence have been acquired by stressing the contribution of the stimulus features, while employing “basic” button press responses. Other studies, instead, attributed a crucial role to the type of motor responses in eliciting spatial correspondence, or grasping affordance mechanisms. These studies displayed that motor affordances are more likely to emerge if instructed responses resemble those typically associated with object stimuli (Couth et al., 2014). For instance, Pavese and Buxbaum (2002) demonstrated that a graspable distracter slowed RTs for reaching and grasping responses more than for button press ones. Bub and Masson (2010) displayed that a reach and grasp action yielded alignment effects with handles, while none were found for a button-press response (see also Bub, Masson, & Kumar, 2018). More recently, Bub Masson and van Noordenne (2021) provided evidence that limb-specific, in place of response location-specific, effects on response selection can be generated automatically by the task-irrelevant image of a graspable object. They observed handle-to-hand alignment effects either with keypress or reach-and-grasp responses to the laterality of a hand picture superimposed on an object picture. In both cases, control processes implicated in the planning of reach-and grasp actions were recruited: these processes ultimately determined the task-irrelevant object to trigger motor features rather than spatial ones (but see Pellicano et al., 2019, and Pellicano & Binkofski, 2021 for evidence of basic spatial correspondence effects with precision grasp and reach-and-grasp responses modelled on the identity and the size of tool stimuli).

Other studies supported the affordance activation hypothesis utilizing S-R correspondence paradigms similar to ours, with tool-object pairs mapped to button press responses (Colman et al., 2017; Borghi et al., 2012). Differently from us, these studies further enriched the tool-object visual scene by adding a hand picture stimulus close to the active tool and obtaining grasping potentiation effects for the dominant right hand.

In the present study we kept our interest focused on the spatial codes produced by tool stimuli, and in particular, on the kind of spatial coding mechanisms involved in the processing of tool-object configurations mapped to button press responses, and responsible for object-based spatial correspondence effects.

In the present investigation, both the experiments showed no support to our hypothesis that the coding of action directions, linking together tools and objects, should be favored at most by concurrent semantic relation and correct alignment for action. Instead, tool-object relations had separate, independent effects on performance. If on the one hand we could not reconcile the present results with our original interpretation (Pellicano et al., 2017), on the other hand the present scenario was substantial and suggested a plausible, alternative explanation that involved *perceptual grouping* mechanisms applied to common use objects (see Gronau et al., 2008; Kaiser et al., 2014).

Accordingly, objects and tools should not be considered as stand-alone entities, but as embedded in typical contextual settings whose regularity facilitates their visual processing (see Bar, 2004 for a review). Several sources of information can contribute to set-up recognizable contexts, for example: identity-related (semantic) and location-related (spatial) information can contribute to group together objects when they share such features relative to when they do not (Gronau et al., 2008). Kaiser Stein and Peelen (2014) provided behavioral and fMRI evidence that the human visual system exploits long-term visual experience to group objects that typically co-occur in certain configurations. In visual search experiments, participants detected a target object between pairs of distracters, either in their typical co-occurrence (e.g., a lamp above a table) or in an irregular configuration (e.g., a lamp below a table). Target object detection was improved when distracter objects were positioned according to real-world regularities. Indeed, their grouping reduced the competition for neural representation and resulted in more efficient visual perception.

When considered in the light of such logical framework, our results suggested that one grouping mechanism followed the processing of semantic, or alignment relations between the tools and the objects, and preceded (allowing for) the basic spatial coding of visual stimuli, which in turn determined the S-R correspondence effect. Specifically, tool-object pictures were perceptually grouped each time based on their task-relevant feature.

In Experiment 1, once a semantic relation was overtly detected (e.g. creamer + cup), this worked as a grouping criterion between tools and objects. Consequently, the different positioning of the side object relative to the central tool became a source of visual asymmetry that was spatially coded (i.e., making the observer aware of left- and rightward jutting tool-object pairs). In turn, response selection was facilitated when the correct response shared the same spatial code as the stimulus or delayed when stimulus and response had opposite spatial codes. Along with the same logic, when tools and objects shared no semantic relation (e.g. creamer + nail), they were not perceptually grouped, so that no lateral asymmetry could be detected, and no correspondence effect could be produced (Figure 2, upper panel).

Similarly, in Experiment 2, tool-object pictures were perceptually grouped based on their task-relevant alignment that allowed for their spatial coding and, in turn, for the S-R spatial correspondence effect. Instead, when tools and objects resulted misaligned, again no grouping into one stimulus configuration, no lateral orientation, and no correspondence effect could take place (Figure 3 lower panel). To complete the picture, the significant action × correspondence × experiment interaction for ERs put in evidence that in Experiment 2 performance was numerically less error prone in corresponding relative to non-corresponding trials when tools and objects were aligned. However, the pattern was opposite and significant when they were misaligned. This suggested that – provided that tool-object misalignment brought no grouping and ultimately to no coding of macroscopic asymmetry – the asymmetry of the tool alone biased to some extent the accuracy of performance by inducing the spatial coding of its protruding side. Since this side pointed in the opposite direction as the object location (see Figure 1), the resulting correspondence effect reversed its sign (Figure 3, lower panel).

We also considered an alternative explanation for the results of experiment 2, according to which correspondence effects observed with aligned pairs would not depend on the global configuration of the tool together with its object (i.e., their perceptual grouping), but on the tool alone, with no actual grouping. Such alternative interpretation, however, would hardly conceal with the significant action × experiment interaction, for which, in experiment 2, performance was faster when tools and objects were aligned relative to when they were misaligned. This effect, followed by the significant interactions of correspondence with action and experiment for both RTs and ERs, suggested that participants did attend to the identity of both the central tool and the lateral object, making rather implausible that, in the aligned condition, participants neglected the side object at all, to only focus on the tool orientation. Then, tools and objects were grouped together, or they did not, according to the presence or absence of alignment, respectively. At that point, any emerging evidence of location coding based on the tool alone (like the one we observed in the action × correspondence × experiment interaction for ERs) would only be subordinate to the lack of perceptual grouping (i.e., in the misaligned condition), which would allow, to some extent, the visual asymmetry provided by the central tool alone to drive the correspondence effect.

Thus, semantic and alignment features acted as grouping factors independently from each other. Specifically, the alignment feature per se was exploited as grouping criterion only when it overtly subserved a response-selection strategy (Experiment 2), whereas it was substantially ignored when it was task-irrelevant (Experiment 1); so, weaker representations of correct alignments were stored in the cognitive system, that were unable to result in automatic processing. Differently, the semantic relation between tools and objects, beyond acting as task-relevant grouping factor (Experiment 1), could not be filtered out when task-irrelevant, and covertly induced perceptual grouping in Experiment 2. This is consistent with the view that semantic representations of pictures, as well as semantic associations between them, can disregard visual contingent details and metric coordinates (e.g., Carr et al., 1982; see also Gronau et al., 2008) relative to representations of correct physical alignments; so that their retrieval was easier.

Lastly, the present results can contribute to the debate on the neurocognitive basis of tool use. The knowledge for visual objects in the human brain has been accounted by a model which includes an indirect, vision-to-perception, semantic route associated with the visual *ventral stream* (VS – occipito-temporal areas: lateral occipital complex LOC, middle temporal MTG, inferior temporal ITG, fusiform gyrus FG), and two direct, vision-to-action, and non-semantic routes: the *ventro-dorsal stream* (VDS – anterior intraparietal sulcus aIPS, ventral premotor cortex PMv) and the *dorso-dorsal stream* (DDS – V6A, medial intraparietal sulcus mIPS, dorsal premotor cortex PMd) (see Binkofski & Buxbaum, 2013). The VS represents objects’ identity and knowledge by accessing semantic memory (Milner, 2017). The DDS contributes to the planning of reaching movements (Davare et al., 2015), while the VDS integrates grasp-related information (Davare et al., 2010).

Within such an established anatomo-functional substrate, a three action-system model – 3AS (Osiurak et al., 2017; Reynaud et al., 2016) has been proposed that offers a comprehensive approach to different object knowledge at *physical* and *neurocognitive* levels (see also Lesourd et al., 2021). Accordingly, one pair of objects allows at a physical level for a tool-to-object mechanical action, when a correct alignment is provided (e.g., a teapot and a cup correctly aligned to each other). This will correspond at neurocognitive level to a mechanical knowledge in the VDS. The same pair would share at a physical level a tool-to-object contextual (semantic) relationship (i.e., two kitchenware), which corresponds at neurocognitive level to a function knowledge along the VS. Lastly, the pair elicits, at physical level, hand-to-tool affordances corresponding, at neurocognitive level, to a motor control system in the DDS (hand grasping the teapot to pour tea in the cup).

Thus, for what concerns our study closely, we speculate that tool-to-object correct alignment representations would depend on the contribution of the VDS, whereas semantic links should derive from VS functionality. Our results well fit within this part of the 3AS model, but also suggest that semantic and alignment relations between tools and objects could be, under certain conditions, independently subserved by VS and VDS with no further crosstalk or integration. This would raise the question whether similar mechanical knowledge (activation patterns) would be produced within the VDS by aligned tool-object pairs irrespective of semantic relation and to what extent VS and VDS functionalities would be involved in the perceptual grouping of tool-object stimuli. These constitute, in our opinion, relevant basic targets for future imaging studies.

In a recent contribution to the investigation on tool use, Federico and Brandimonte (2020) performed two eye-tracking experiments on thematically consistent and -inconsistent tool-object pairs (e.g., whip-bowl and whip-shoe, with the tool always correctly pointing at the object) that were supposed to elicit in the observer higher and lower action readiness, respectively. When participants simply looked at the tool-object pairs (free visual exploration – experiment 1), in the thematically consistent condition, tools were fixated longer on their handle (manipulable part), whereas in the thematically inconsistent condition they were fixated longer in their functional part, opposite to the handle (see also Federico & Brandimonte, 2019). Crucially, when participants looked at the tool-object pairs to lately perform a yes-no recognition task on one item of the pairs (experiment 2), longer fixations in the functional area of the tools were induced, irrespective of the relationship with their objects. In contrast to the embodied cognition approach, the authors proposed a reasoning-based approach to tool use claiming that to interact with the environment, people primarily process semantic (functional) rather than sensorimotor information. Our results would fit within this idea: beyond our evidence that accurate control for basic stimulus asymmetries contributes eliminating handle-to-hand correspondence effects, such a pre-eminence of semantic over sensorimotor processing (Federico & Brandimonte, 2020) could be plausibly taken as a further factor to foster perceptual grouping-location coding mechanisms.

In conclusion, the present study provided evidence for a location coding account of object-based correspondence effects when tool-object pairs were employed as visual stimuli and mapped to button press responses. Semantic and alignment features subserved as perceptual grouping criteria independent from each other and with stronger effects of the former. This allowed for the spatial coding of visual asymmetries, and in turn generated a S-R correspondence effect.

In memory of Robert W. Proctor.

## Data Accessibility Statement

Data are available at the OSF public data repository https://osf.io/x75qh/?view_only=d8e2aee2c90c4cd5877b98521ee575b6.

## Additional File

The additional file for this article can be found as follows:

10.5334/joc.435.s1 Supplementary Material. Supplementary statistical analyses.

## Footnotes

At 57 centimeters of eye-screen distance, 1 cm on the screen corresponds, with good approximation, to 1 degree of visual angle.

Six participants, who did not take part to the experiment, were asked to describe the visual scene offered by misaligned tool-object combinations, while no mention was made of the possible semantic link or action alignment. To note, the tool-object configuration that included the upright hammer with its head tilted away from the upside-down object (homologous of the creamer with its spout oriented away from the upside-down object) was not judged as misaligned, but rather as still aligned, by four out of six participants and therefore prudently discarded from the pool of stimuli.
