# Supplementary material for: Location Coding of Tool-Object Pairs Based on Perceptual Grouping: Evidence from Object-Based Correspondence Effect
Source: J Cogn. 2025 Feb 26;8(1):24. doi: 10.5334/joc.435 (PMC11869823; doi:10.5334/joc.435)
Supplement: Supplementary Material. — Supplementary statistical analyses. [file joc-8-1-435-s1.pdf]

Location Coding of Tool-Object Pairs Based on Perceptual Grouping: Evidence from Object-based Correspondence Effect
– *Supplementary materials* –

Usman Jawed Shaikh[1], Ferdinand Binkofski[1, 2, 3], and Antonello Pellicano[4 *]

[1]*Division for Clinical Cognitive Sciences, Department of Neurology, Faculty of Medicine, RWTH Aachen University, Aachen, Germany,*

[2]*Juelich Aachen Research Alliance (JARA)—BRAIN, Juelich, Germany,*

[3]*Research Centre Juelich, Institute of Neuroscience and Medicine (INM-4), Juelich, Germany.*

[4]*Department of Educational Sciences, University of Catania, Catania (Italy).*

*Corresponding author at:
Department of Educational Sciences
University of Catania
Via Teatro Greco, 84
95124 - Catania
E-Mail: antonio.pellicano@unict.it

Supplementary materials

Analyses on restricted stimulus materials.

It has been observed that the perception of objects identified as dangerous can induce aversive affordances, so that motor responses tend to “escape away” from them. For example, in Anelli, Ranzini, Nicoletti and Borghi (2013) when participants bisected lines flanked by one dangerous and one neutral graspable object, they misperceived the line midpoint toward the neutral graspable object opposite to the dangerous graspable one.

In our set of visual stimuli, we included *nails* as side objects paired with tools (see figure 1). The tool-object arrangements across the semantic category and alignment variables included nails in half the total of tool-object pairs; a quarter of which had upside-down nails.

Since upside-down nails are potentially identifiable as harmful, we wanted to verify whether their perception within tool-object pairs could have biased the motor performance at the S-R correspondence task. Because of their potentially dangerous nature, nails could have afforded manipulative actions (independent of the tool-object pairings at hand) when “safe”, (i.e., upright oriented), and inhibited such actions when harmful (i.e., upside-down).

To evaluate this, we performed a control ANOVA on normalized RTs restricted to the tool-object pairs that included a nail and with the same factors as the full analysis (i.e., experiment x category x action x correspondence). The interactions of interests were the *category x action x correspondence* which turned significant $F(1, 43) = 4.329$, $p = .043$, $\eta_p^2 = .09$, and the nonsignificant *experiment x category x action x correspondence*, $F(1, 43) = 1.334$, $p = .255$, $\eta_p^2 = .03$. The correspondence effect was significant when tool-object pairs were aligned and from the same category (i.e., upright hammer + upright nail), $t(43) = 2.889$, $p = .006$, $d_z = 25.2$, as well as from different categories (i.e., upright creamer + upright nail) $t(43) = 3.043$, $p = .004$, $d_z = 26.08$. Crucially, the correspondence effect was not significant in both misaligned-same (i.e., upside-down hammer + upright nail), $t(43) = 1.591$, $p = .119$, $d_z = 19.52$ and misaligned-

different conditions (i.e., upright creamer + upside-down nail), $t(43) = 2.028$, $p = .049$, d_z = 21.73 (Bonferroni-corrected alpha = .0125). If the safe nail object afforded manipulative actions, which were inhibited when the same nail became dangerous, then one would expect a significant S-R correspondence effect in the misaligned condition with safe nails, but no effect in the misaligned condition with harmful nails, because of no S-R corresponding response facilitation due to inhibition of manipulative actions. Our results instead displayed that correspondence effect was absent, in the misaligned condition, either when an upside-down harmful nail was displayed as side object, or when the same nail was upright and "safe". In our opinion these results hardly suggest an affordance activation/inhibition account, but rather they look consistent with our original interpretation in terms of location coding based on perceptual grouping.

Block analysis.

In our experiments we created tool-object stimuli by pairing each of the 6 tools with 4 objects and arranged according to same-different category, action alignment-misalignment, and leftward and rightward orientation, for a total of 96 stimuli, which were repeated 6 times across 3 blocks. Given the relatively hight repetition of stimuli across the experiments, we controlled for possible effect of trial blocks on the effects of interest.

We performed an ANOVA on normalized RTs, adding a Block (block1 vs. block2 vs. block3) within-subjects factor, to the statistical model of the principal analyses. We observed a main effect of *block* and significant *block x category* and *block x action x correspondence* interactions. The overall speed of performance increased progressively across successive blocks (block 1= 292 ms, block 2 = 279 ms, block 3 = 275 ms), $F(2, 86) = 29.461$, $p < .001$, $\eta_p^2 = .41$. The difference between different and same category conditions decreased across successive blocks (block 1 = 13ms, block 2 = 12ms, block 3 = 8ms), $F(2, 86) = 4.151$, $p = .019$, $\eta_p^2 = .08$. The correspondence effect in the aligned condition decreased in size across successive blocks (block 1 = 9ms block 2 = 5ms, block 3 = 4ms), whereas it remained nonsignificant in the misaligned

condition of the three blocks (block 1 = 2ms block 2 = 2ms, block 3 = 3ms), $F(2, 86) = 6.301$, $p = .003$, $\eta_p^2 = .13$. These results, in our opinion, illustrate a basic effect of practice that - across increasing trials - speeded-up performance, reduced the advantage in the processing of pairs from same categories relative to pairs from different categories, and most of all, reduced the interference of S-R noncorresponding trials in aligned tool-object pairs. These last results are basically consistent with evidence of general practice effects in S-R compatibility literature (e.g., Dutta & Proctor, 1992; Proctor & Lu, 1999) but had no impact on the effects so far discussed.

Hands analyses.

With these analyses we wanted to explore the possible effects of responding hands on the location coding of our tool-object pairs, and verify whether grasping affordances could have been elicited, to some extent, for the dominant right-hand responses, only. Past studies provided evidence of affordance activations effects within enhanced visual context given by pictures of tool-object pairs mapped to right hand responses (see Borghi et al., 2012; Yoon et al., 2010). Further, in Colman, Remington and Kritikos (2017) left and right handed participants completed a covert visual cueing task, while discriminating between two target shapes. They provided evidence that biased visuospatial attention enhanced object identity discrimination near hands, but crucially these effects resulted particularly enhanced for right-handers.

We performed separate ANOVAs on normalized RT and Error rates data of Experiment 1 and Experiment 2, adding the between-participant Mapping factor (“A” vs. “B”). In experiment 1, eleven participants pressed the left button to same category and the right button to different categories conditions (mapping A). Conversely, the other eleven participants pressed the right button to same category and the left button to different categories conditions (mapping B). In experiment 2, eleven participants with mapping “A” pressed the left button to aligned tools and objects, and the right button to misaligned ones; participants assigned to mapping “B” pressed

the right button to aligned, and the left button to misaligned pairs. For both the experiments, the statistical model was Mapping x Category x Action x Correspondence.

For RTs, the main effect of mapping was not significant in both experiment 1, $F(1, 20) = 0.362$, $p = .554$, $\eta_p^2 = .02$, and experiment 2, $F(1, 20) = 1.920$, $p = .181$, $\eta_p^2 = .09$. Among the informative interactions, in experiment 1 we replicated the significant category x correspondence we obtained in the principal analyses, $F(1, 20) = 15.351$, $p < .001$, $\eta_p^2 = .43$. We displayed a significant correspondence effect between the protruding side of tool-object pairs and the responding hand in the same category condition, $t(21) = 3.109$, $p = .005$, $d_z = 12.91$, but not in the different category one, $t(21) = 1.713$, $p = .101$, $d_z = 26.08$ (Bonferroni-corrected alpha = .025); however we observed a nonsignificant mapping x category x correspondence interaction, $F(1, 20) = 0.918$, $p = .349$, $\eta_p^2 = .04$, that is, the correspondence effect observed in the same category condition did not change between the right (6 ms in size, mapping A) and the left hand performance (11 ms, mapping B).

In experiment 2, we replicated the significant Action x Correspondence interaction, $F(1, 20) = 22.589$, $p < .001$, $\eta_p^2 = .53$, with a significant protruding side to hand correspondence effect in the aligned, $t(21) = 4.550$, $p < .001$, $d_z = 23.67$, but not in the misaligned condition, $t(21) = 0.157$, $p = .877$, $d_z = 15.69$ (Bonferroni-corrected alpha = .025). We observed a significant Mapping x Action x Correspondence interaction, $F(1, 20) = 7.149$, $p = .015$, $\eta_p^2 = .26$, due to a significant correspondence effect in the aligned condition mapped to the left hand (29 ms in size, mapping A), $t(10) = 5.207$, $p < .001$, $d_z = 18.73$, and a nonsignificant effect in the aligned condition mapped to the right hand (16 ms in size, mapping B), $t(10) = 2.021$, $p = .071$, $d_z = 27.09$, although consistent with the size of correspondence effects in Pellicano et al. (2017).

For ERs, main performance did not differ between the two mappings in both experiment 1, $F(1, 20) = 0.551$, $p = .466$, $\eta_p^2 = .03$, and experiment 2, $F(1, 20) = 1.134$, $p = .300$, $\eta_p^2 = .05$. We only observed, in experiment 1, a “weak” interaction between mapping and category, $F(1, 20) = 4.493$, $p = .047$, $\eta_p^2 = .18$, with some larger difference between same and different categories

conditions in mapping A relative to mapping B, even though post-hoc tests did not show significances, *ts*(10) < 1.0.

Results were in line with those of the principal analyses in suggesting that a perceptual grouping mechanism allowed the relative positioning of tools and objects to become a source of visual asymmetry. Such asymmetry was spatially coded, and a S-R correspondence effect was driven by the location of the protruding side of the tool-object pairs. The correspondence effects had the same positive sign in both the experiments for both the responding hands. However, in experiment 2, the correspondence effect was smaller and nonsignificant in the aligned condition mapped to the right dominant hand, although its size was consistent with the ones observed in the original Pellicano et al. (2017) investigation. This could be attributable to a type-II error, due to the number of participants inevitably halved because of the between-participant mapping variable, and the consequent reduction of statistical power. However, net of these limitations, we can only speculate that the spatial coding of the protruding side of the stimulus was weakened by some emerging concurrent affordance of the opposite graspable side. Thus, results might offer some indications that the dominant right-hand performance, which was assumed to be more naturally prone to skilled manipulations-use of tools, could to some extent support the activation of grasping affordances. This however stays as a cautious interpretation, that would deserve stronger evidence in future experiments. To note, different from the present study, previous studies that obtained stronger grasping potentiation effects for the dominant right hand, also enriched the visual scene by implementing a hand picture stimulus close to the active tool (Colman et al., 2017; Borghi et al., 2012; Yoon et al., 2010; see also Bub et al., 2021). Future systematic follow-up investigations would clarify whether a boundary between spatial coding and affordance mechanism can be identified through increased complexity of the visual context from the perceptual and motor point of view.

References

Anelli, F., Ranzini, M., Nicoletti, R., & Borghi, A. M. (2013). Perceiving object dangerousness: an escape from pain? Experimental Brain Research, 228, 457-466. https://doi.org/10.1007/s00221-013-3577-2

Borghi, A. M., Flumini, A., Natraj, N., & Wheaton, L. A. (2012). One hand, two objects: Emergence of affordance in contexts. Brain and Cognition, 80(1), 64-73. https://doi.org/10.1016/j.bandc.2012.04.007

Colman, H. A., Remington, R. W., Kritikos, A. (2017). Handedness and Graspability Modify Shifts of Visuospatial Attention to Near-Hand Objects. PLoS ONE 12(1): e0170542. https://doi.org/10.1371/journal.pone.0170542

Dutta, A., & Proctor, R. W. (1992). Persistence of stimulus–response compatibility effects with extended practice. Journal of Experimental Psychology: Learning, Memory, and Cognition, 18, 801–809.

Proctor, R. W., & Lu, C. H. (1999). Processing irrelevant location information: Practice and transfer effects in choice-reaction tasks. Memory & Cognition, 27, 63-77.

Yoon, E. Y., Humphreys, G. W., & Riddoch, M. J. (2010). The paired-object affordance effect. Journal of Experimental Psychology: Human Perception and Performance, 36(4), 812–824. https://doi.org/10.1037/A0017175
